# Supplementary material for: Botulinum Toxin Type A (BoNT-A) Use for Post-Stroke Spasticity: A Multicenter Study Using Natural Language Processing and Machine Learning
Source: Toxins (Basel). 2024 Aug 2;16(8):340. doi: 10.3390/toxins16080340 (PMC11359065; doi:10.3390/toxins16080340)
Supplement: Supplementary file 1 [file toxins-16-00340-s001.zip › toxins-3059622-supplementary.pdf]

Botulinum Toxin Type A (BoNT-A) Use for Post-Stroke Spasticity: A Multicenter Study Using Natural Language Processing and Machine Learning

SUPPLEMENTAL METHODS

Extraction of the Unstructured Information from Electronic Health Records

All study variables were extracted from patients' Electronic Health Records (EHRs) using EHRead® technology which uses Natural language processing (NLP) and Machine learning (ML) techniques for extracting and translating free text into a study database. This process required that conceptual definitions for all study variables were pre-specified and aligned with clinical entities found in the SNOMED Clinical Terms (a comprehensive, computationally processable collection of medical terms utilized in clinical documentation) using the SNOMED CT browser. This step facilitated the conversion of unstructured data from various hospital departments into actionable variables for extraction. The clinical accuracy of the conceptual definitions and entity mapping was reviewed and approved by medical research experts specialized in NLP.

Once the clinical entities were extracted, variables were constructed by applying dedicated data wrangling operations to their mapped entities, leveraging specific NLP parameters generated by dedicated ML models (e.g., negation, temporality, attributes, etc.) and record-specific metadata (e.g., date, medical department, record type, etc.).

EHRead® performance

To ensure the quality of data extraction, the performance of EHRead® was externally evaluated. Specifically, this validation was carried out by external annotators following a peer-reviewed method [1]. Briefly, the external annotators created the ‘standard’ to which EHRead® technology’s variable detections were compared. The aim was to measure inter-annotator

agreement (IAA) to ensure guideline consistency and parameter reliability, using these annotations as a benchmark to assess EHRead® against physician annotations.

Additionally, to determine the required minimum number of annotated EHRs, we employed the Sample Calculator for Evaluation (SLiCE®), a tool designed to calculate this based on the prevalence of key variables (in this case, Post-stroke spasticity and botulinum neurotoxin type A) within the EHRs. SLiCE uses a 95% confidence level, interval widths of 10% (percentage points), and targets for precision and recall, ensuring that the estimated precision and recall are accurate within ±5% (percentage points) at a 95% confidence level.

The evaluation of the system was calculated in terms of the standard metrics of Precision, Recall, and their harmonic mean F1-Score.

- *Precision* = $\frac{tp}{tp + fp}$. This parameter indicates the accuracy of the system in retrieving key clinical concepts.
- *Recall* = $\frac{tp}{tp+fn}$. This parameter indicates the amount of information the system retrieves.
- *F1-Score* = $\frac{2xPrecisionxRecall}{Precision+Recall}$. This parameter gives us an overall performance indicator of information retrieval.

In all cases, *tp* is the number of true positives (i.e., records correctly retrieved), *fn* is the set of false negatives (i.e., records incorrectly not retrieved), and *fp* is the number of false positives (i.e., records incorrectly retrieved).

REFERENCES

1. Canales, L., et al., *Assessing the Performance of Clinical Natural Language Processing Systems: Development of an Evaluation Methodology.* JMIR Med Inform, 2021. **9**(7): p. e20492.
